# An arc fault diagnosis algorithm using multiinformation fusion and support vector machines

**DOI:** 10.1098/rsos.180160

**Published:** 2018-09-19

**Authors:** Jian-hong Yang, Huai-ying Fang, Ren-cheng Zhang, Kai Yang

**Affiliations:** Key Laboratory of Process Monitoring and System Optimization for Mechanical and Electrical Equipment (Huaqiao University), Fujian Province University, Xiamen, Fujian 361021, People's Republic of China

**Keywords:** arc fault, high-frequency radiation characteristics, slope characteristics, periodic integration characteristics, multiinformation fusion

## Abstract

Arc faults in low-voltage electrical circuits are the main hidden cause of electric fires. Accurate identification of arc faults is essential for safe power consumption. In this paper, a detection algorithm for arc faults is tested in a low-voltage circuit. With capacitance coupling and a logarithmic detector, the high-frequency radiation characteristics of arc faults can be extracted. A rapid method for computing the current waveform slope characteristics of an arc fault provides another characteristic. Current waveform periodic integral characteristics can be extracted according to asymmetries of the arc faults. These three characteristics are used to develop a detection algorithm of arc faults based on multiinformation fusion and support vector machine learning models. The tests indicated that for series arc faults with single and combination loads and for parallel arc faults between metallic contacts and along carbonization paths, the recognition algorithm could effectively avoid the problems of crosstalk and signal loss during arc fault detection.

## 1. Introduction

Electrical safety is critical for everyday life. Electric circuits are usually installed at hidden locations. Early arc faults are difficult to detect because of weak signals and less obvious fault characteristics. Such arc faults may lead to fires, which pose a great threat to lives and property [1–3]. For electrical fires caused by short circuits of arc faults, traditional electric circuit protection devices cannot be relied upon for early detection. Most methods for detecting arc faults are based on measurement of the state characteristics of the circuit current [4]. Typical diagnostic methods include frequency domain analyses [5], time domain waveform analyses [6], analyses of autoregressive model parameters [7] and high-order spectral analyses [8]. Two fast arc fault detection methods have been proposed in this paper with the analysis of only half cycle data. Both fast Fourier transform and wavelet packet decomposition have been adopted to distinguish arc fault currents from normal operation currents. Analysis results show that alternating current arcs can be effectively and accurately detected with the proposed half cycle data-based methods [9]. Wu & Liu employ the discrete wavelet transform and an artificial neural network to identify the occurrence of serial arc faults on indoor low-voltage power lines [10]. Jovanovic *et al*. present a novel method based on a single-phase active power filter for series arc fault detection in an AC electrical installation. This method's reference current is used as the starting point on a large variety of loads: resistors, vacuum cleaner, rotary drill, dimmer and AC–DC power supplies [11]. But, this method is not suitable for the identification of parallel arc faults. For arc fault identification, these analytical methods are effective in specific electrical circuits or working conditions, but some of the characteristics are affected by load power, circuit breakage, nonlinear loads and other factors. These loads are constantly disconnected and connected. Therefore, loads in typical power lines are complicated and dynamic, which presents challenges for these static methods. In addition, electrical circuits usually have impedances and capacitor filters that suppress the characteristic signal for arc faults. Therefore, a single characteristic signal in a circuit current cannot effectively detect arc faults. The above-mentioned recognition algorithm of arc faults can miss or misjudge detection [12]. Studying the influence of circuit characteristics on the suppression of different arc fault signals will help in detecting arc faults more accurately.

High-frequency radiation characteristics (HFRCs) can be easily detected for series arc faults caused by carbonization path, capacitance filter suppression and line impedance suppression. However, the HFRC signal of the series arc faults in adjacent loop will disturb the normal circuit. For the parallel arc faults caused by the carbonization path and metal contact, the HFRC of the arc faults caused by the metal contact is very weak, and it is easy to lose the arc half wave. Therefore, it is not reliable in detecting series and parallel arc faults accurately. When the electrical load is simple, the current waveform slope characteristics (CWSCs) in series and parallel arc faults are easy to detect. The CWSC is very complex, which is easily affected by the amplitude of current. Some calculation methods are not affected by the amplitude of the current. However, the load of the electrical circuit is complex, and the CWSC is easily influenced by the inhibitory load (switch power, for example). For some nonlinear loads, current waveform periodic integral characteristics (CWPICs) of arc faults are easily changed, but the CWPIC of the arc faults is easily affected by the load starting current.

The arc faults can be identified under specific conditions by HFRC, CWSC and CWPIC. But, there is some limitation in the identification of arc faults with one of the characteristic signals. In this paper, the simple extraction methods of three characteristic signals are studied and can be easily implemented in a single-chip microcomputer system. We developed an experimental method to investigate capacitor filter suppression and impedance suppression of arc fault signals. In a model circuit with a variety of loads in different operating conditions, we are able to extract three separate characteristics of arc faults. Model parameters of support vector machines (SVM) are optimized, and arc faults are identified by using a multiinformation fusion (MIF) algorithm. The three characteristic signals are fused and the characteristics are compensated by each other. This can avoid the leakage and misjudgement in the process of arc fault recognition.

## 2. Methods

### 2.1. Experimental scheme

**Figure 1.**
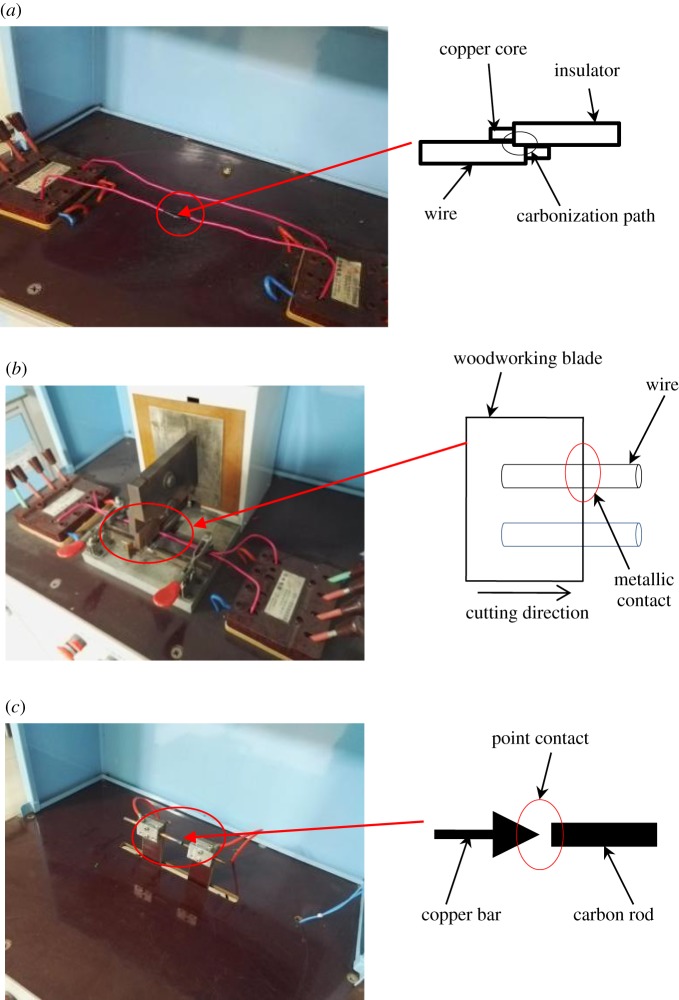
Simulation test device of the arc faults. (*a*) Carbonization path, (*b*) metallic contact and (*c*) point contact.

There are various reasons for arc faults in electrical circuits. Testing standard of arc fault circuit interrupters was set up by Underwriters Laboratories Inc. (UL1699-2011) and China National Standards Committee (GB14287.4-2014). The GB14287.4-2014 is a testing standard of arc fault detection devices under China's electric power environment. The method and working conditions of simulation arc fault are put forward in GB14287.4-2014. An experimental platform for arc fault simulation is built, which simulates series and parallel arc faults by point contact, metallic contact and carbonization path. Some of the loads are selected by UL1699-2011 and GB14287.4-2014. If there are 8 half waveforms of arc in 1 s, the arc faults are considered by UL1699-2011. If there are 12 half waveforms of arc in 1 s, the arc faults are considered by GB14287.4-2014. If there are 12 half waveforms of arc in 1 s, the arc faults are considered in the paper. An arc fault simulation apparatus is shown in figure 1. Figure 1*a* depicts the carbonization path test platform, which was used to simulate arc faults caused by ageing lines or poor contact. Figure 1*b* depicts the metal contact test platform, which was used to simulate arc faults that are caused by contact between insulated metal wires in actual lines, and the contact eventually leading to wire damage. Figure 1*c* illustrates the point-contact arc test platform, which was used to simulate arc faults between insulation and copper wire after carbonization had taken place.

**Figure 2.**
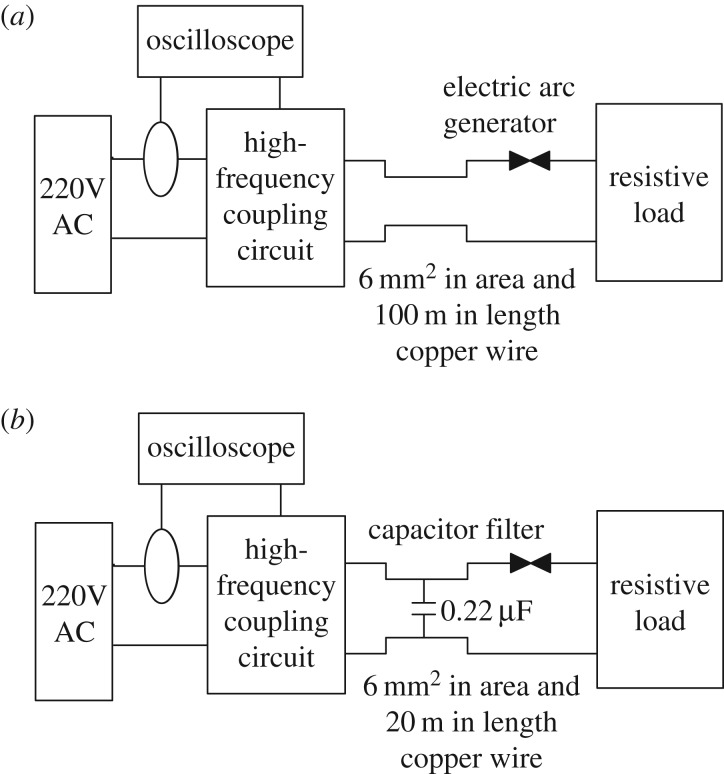
Experimental scheme for load suppression. (*a*) Line impedance suppression and (*b*) capacitor filter suppression.

The load-suppression experimental set-up is shown in figure 2. The power supply of experiments is 220 V AC (50 Hz). Figure 2*a* depicts the circuit used to test impedance suppression of point contact arc fault signals. A resistive load is connected to the experimental circuit. A 100 m long copper wire (surface area: 6 mm^2^) is connected in series between the data acquisition system (Tektronix DPO4104-L) and the arc fault generator. At the same time, HFRC signals are collected at the end near the arc faults. Figure 2*b* is a diagram of the test bed for point-contact arc capacitance filter suppression. Between the high-frequency receiver circuit and the arc generator, a 0.22 µF filter capacitor is inserted to simulate the actual distributed capacitance of the wire. A resistive load and a copper wire 20 m in length are also connected to the circuit.

We found that a distributed capacitance appears between a live line and the ground line in longer circuits, which causes attenuation of the characteristic signal of the arc faults. The effective resistance of the conductor increases because of the skin effect, and the circuit impedance strongly inhibits the HFRC of the arc faults.

### 2.2. Feature extraction of arc faults

#### 2.2.1. HFRC extraction of arc faults

**Figure 3.**
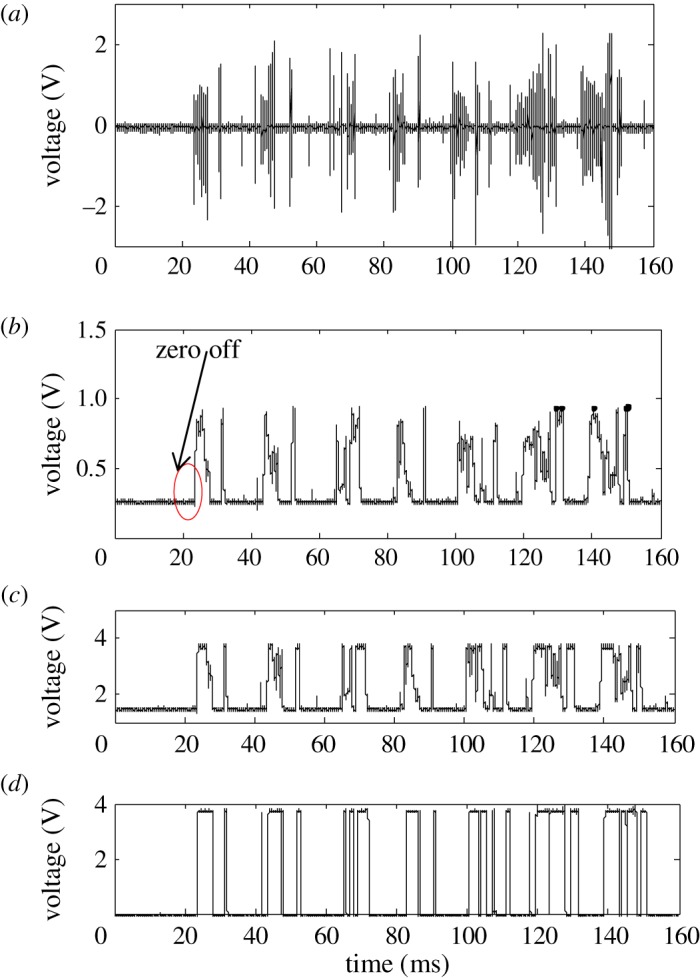
The HFRC extraction method of arc faults. (*a*) The original HFRC signal of the arc faults, (*b*) logarithmic detection signal, (c) linear amplified signal and (*d*) the corresponding pulse signal.

When an arc fault occurs, the amplitude of the HFRC signals greatly varies, so a logarithmic amplifier is needed for nonlinear compression. Figure 3*a* depicts observed HFRC signals of arc faults, and figure 3*b* depicts a logarithmic detection signal of arc faults. As arc faults show the ‘zero off' phenomenon, the amplitude of the arc fault signal given by logarithmic detection fluctuates between 0.25 and 1 V. To improve the voltage gain, which increases the load capacity of the system's back-end and improves anti-jamming performance, the detection signal is linearly amplified. The amplified result is shown in figure 3*c*. For practical applications, the signal is converted to a pulse signal to reduce the microprocessor computation time as shown in figure 3*d*.

#### 2.2.2. CWSC extraction of arc faults

When an arc fault occurs, the CWSC will rise or fall rapidly after the zero crossing. The change of the CWSC can be used as an additional identification criterion for arc faults [12,13]. CWSC extraction algorithm collects *m* data points (*x*_1_, *x*_2_, … , *x_m_*) for the *n*th half wave. Subsequently, the algorithm estimates the CWSC corresponding to the half wave using the following equation:

2.1 $$W_{n}=\frac{{\left(|x_{1}-x_{2}|,\;|x_{2}-x_{3}|,\cdot \cdot \cdot ,\;|x_{m-1}-x_{m}|\right)}_{\max}}{\sum_{a=1}^{m}x_{a}}.$$

In the equation, (|*x*_1_ − *x*_2_|, |*x*_2_ − *x*_3_|, … , |*x_m_*_−1_ − *x_m_*|)_max_ represents the maximum difference between adjacent sample data points, and $\sum_{a=1}^{m}x_{a}$ is the estimated half wave integral value. *W_n_* is the normalized CWSC of arc faults, *m* is the sequence number of the sampled data and *n* is the half wavenumber of the current waveform.

To effectively distinguish an arc fault through the CWSC, the CWSC of *J* consecutive arc currents can be summed and then used as a judgement criterion for the waveform slope. The sum CWSC in equation (2.2) is then a relevant criterion for detecting arc faults:

2.2 $$X_{b}=\overset{J}{\underset{n=1}{\sum }}W_{n}.$$

In the equation, *J* is the half wavenumber of the current waveform. *X_b_* is the sum of the half wave slope of the *J* current waveform.

#### 2.2.3. CWPIC extraction of arc faults

Under the excitation of an AC power supply, the current waveform should periodically appear when the load is in stable operation. In the event of an arc fault, the violent discharge of the arc destabilizes the circuit, resulting in irregularities in the current waveform. Arc faults can therefore be detected by CWPIC. Considering that the positive and negative half waves of the current will be asymmetrical under the normal working condition of loads, we can calculate the current integral cycle from two current half waves using the following equation to calculate the *n*th current-cycle integration value. The current cycle is 20 ms:

2.3 $$I_{m}=\sum_{a=1}^{m}x_{a},$$

where *I_m_* is the *m* cycle integral value and *X_a_* is a sample value of the current waveform.

Therefore, change of the period-integral *X_c_* in the following equation can be used as a criterion for detecting arc faults:

2.4 $$X_{c}=\overset{N}{\underset{\;j=1}{\sum }}|I_{j}-I_{\;j-1}|\;,$$

where *I_j_* is the integral value of the half wave of the *j* current. *N* is a half wavenumber of current. *X_c_* is the sum of the N-CWPIC.

### 2.3. Arc fault identification algorithm

#### 2.3.1. Arc fault recognition algorithm by support vector machines

The HFRC, CWSC and CWPIC indicate arc faults in different circumstances. With this diversity of characteristics, SVM learning models with many information sources were used to construct a recognition algorithm of arc faults.

SVM is a machine learning method based on statistical learning theory and structural risk minimization [14–16]. The algorithm essentially finds a maximum-margin hyper-plane (in a three-dimensional space, its hyper-plane is a two-dimensional plane) to maximize the distance between the hyper-plane and the nearest data point. For given training data points of the form (*x_i_, y_i_*), *i* = 1, 2, … , *n*, *x_i_* is the training point, *y_i_* is the classification label (either 1 or −1) and *n* is the number of training samples. The SVM classification algorithm can be described using the following equation:

2.5 $$\begin{array}{rl} & \min\left(\frac{1}{2}{∥\omega ∥}^{2}+C\sum_{i}^{n}\sigma_{i}\right)\\ & s.t.\;y_{i}[(\omega \;∙\;x_{i})+b]\geq 1-\sigma_{i}, \end{array}$$

where *ω* is the weight vector, *b* is the bias, *σ* is the slack variable and *C* is the penalty factor.

If the samples are linear-separable, then the decision function can be calculated with the following equation:

2.6 $$f\;(x)=\text{sgn}\left\{\sum_{i}^{k}\alpha_{i}^{*}y_{i}(x_{i}\;∙\;x)+b^{*}\right\},$$

where $\alpha_{i}^{*}$ is Lagrange coefficient, b* is Lagrange threshold and be calculated by the equation $[(\omega \;∙\;x_{i})+b]=1-\sigma_{i}$, *k* is the support vector number, *x_i_* is the *i* feature vector, *x* is the total eigenvector and sgn(·) is a symbolic function.

If the samples are linear-inseparable, the nonlinear mapping function *Φ*(·) can be used to map samples from the original space to the high-dimensional feature space, and the optimal classification surface can then be obtained in the high-dimensional feature space. The inner product operation is calculated in this space using the following equation:

2.7 $$K(x_{i},x_{j})=\Phi (x_{i})\;∙\;\Phi (x_{j}).$$

The inner product function *K*(*x_i_*, *x_j_*) can be used for linear classification after the nonlinear transformations.

#### 2.3.2. Kernel parameter optimization calculation for support vector machine

The inner product function *K*(*x_i_*, *x_j_*) affects the classification results. Different inner product functions can yield different algorithms. Frequently used inner product functions include the linear kernel function, the polynomial kernel function, the radial basis kernel function and the sigmoid kernel function. Of these, the polynomial kernel function and the radial basis function only involve one parameter. The parameter optimization calculations are simple. Therefore, we chose the polynomial kernel function as the inner product function. In [17] and [18], five kinds of upper-bound algorithms are introduced. We used the radius-spacing (RM boundary) upper-bound algorithm to express the kernel function parameters that need to be optimized with *β*. *K*(*x_i_*, *x_j_*) is then a function of *β*. The kernel function can be expressed as *K_β_*(*x_i_*, *x_j_*). Given the range (*β*_min_, *β*_max_) in which *β* needs to be optimized, we substitute *β*_min_ into the following equation as the initial value:

2.8 $$P(\alpha )=\sum_{i=1}^{n}\alpha_{i}-\frac{1}{2}\sum_{i=1}^{n}\sum_{\;j=1}^{n}\alpha_{i}\alpha_{j}y_{i}y_{j}K_{\beta }(x_{i},\;x_{j}).$$

The optimization coefficient $\alpha_{i}^{0}$ can be calculated from the equation. Substitute *β*_min_ as the initial value into the following equation:

2.9 $$∥w{∥}^{2}=2\sum_{i=1}^{n}\alpha_{i}^{0}-\sum_{i=1}^{n}\sum_{\;j=1}^{n}\alpha_{i}^{0}\alpha_{j}^{0}y_{i}y_{j}K_{\beta }(x_{i},\;x_{j}).$$

*w*^2^ can be calculated by the equation. The optimization coefficient $\theta_{i}^{0}$ can be obtained by the following equation:

2.10 $$\begin{array}{rl} R^{2}(\theta ) & =\max\left(\sum_{i=1}^{n}\theta_{i}K_{\beta }(x_{i},\;x_{i})-\sum_{i,j=1}^{n}\theta_{i}\theta_{j}K_{\beta }(x_{i},\;x_{j})\right)\\ \;\text{and}\;\sum_{i}^{n}\theta_{i} & =1,\;\theta_{i}\geq 0\;i=1,\;2,\;\ldots ,\;n. \end{array}\}$$

*R*^2^ can be obtained by the following equation. *R* is the minimum sphere radius, which contains the data of the feature space:

2.11 $$R^{2}=\sum_{i=1}^{n}\theta_{i}^{0}K_{\beta }(x_{i},\;x_{i})-\sum_{i,j=1}^{n}\theta_{i}^{0}\theta_{j}^{0}K_{\beta }(x_{i},\;x_{i}).$$

Substituting ||*w*^2^|| and *R*^2^ obtained from equation (2.9) into equation (2.12), the SVM generalization error metric parameter *T* is obtained.

2.12 $$T=\frac{1}{n}R^{2}∥w∥{}^{2}.$$

*C* is a penalty factor that represents tolerance for recognition errors, where *C* takes different values, identifies each classifier and determines a suitable penalty factor. The optimization steps for *β* and *T*^0^ are as follows: first, the optimization coefficient $\theta_{i}^{0}$ can be obtained by equation (2.10). Second, *R*^2^ can be calculated by equation (2.11). Third, ||*w*||^2^ can be calculated by equation (2.8) and equation (2.9). Finally, *T* can be calculated by equation (2.12). When *T* takes the minimum, the optimal *β* value and *T*^0^ value can be calculated.

#### 2.3.3. Multiinformation fusion diagnosis of arc faults

**Figure 4.**
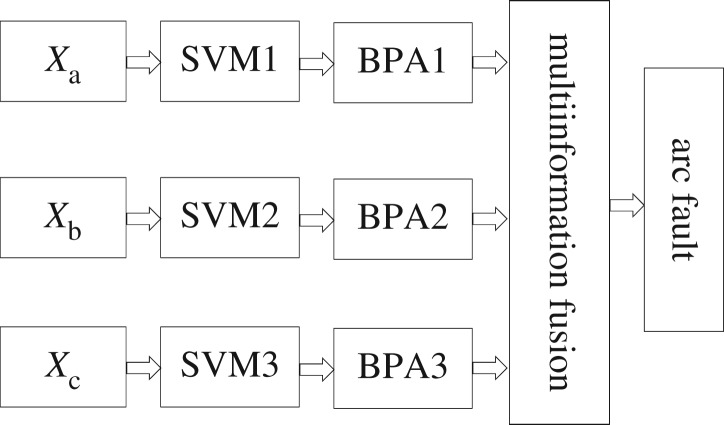
Recognition algorithm of arc faults.

We input the HFRC *X_a_*, the CWSC *X_b_* and the CWPIC *X_c_* during arc faults and normal operation into the SVM model. Considering the fitting and forecasting ability of samples comprehensively, by multiple tests and calculations, specify *C* = 100. Then, the SVM parameters are optimized to *β* = 3 and *T*^0^ = 4.1. Three SVM models, SVM1, SVM2 and SVM3, are obtained after training. The three SVM models output the basic probability assignment (BPA) that is required for MIF, and there are two possible identification results of each SVM model. So, a 3 × 2 BPA matrix is obtained. After normalization, the matrix element sum of every row becomes 1. In this matrix, we multiply one row transposition with another row. After that, a new 2 × 2 matrix R is obtained. The main diagonal elements of matrix R are the cumulative factors of BPA. The sum of the non-main diagonal elements constitutes the uncertainty factor of the evidence. According to the Dempster–Shafer evidence theory [19], the multilayer fusion algorithm based on matrix analysis fused the three streams of characteristic information to identify the arc faults. A scheme of arc fault identification algorithm is shown in figure 4.

## 3. Results and discussion

### 3.1. Arc fault identification by the HFRC

**Figure 5.**
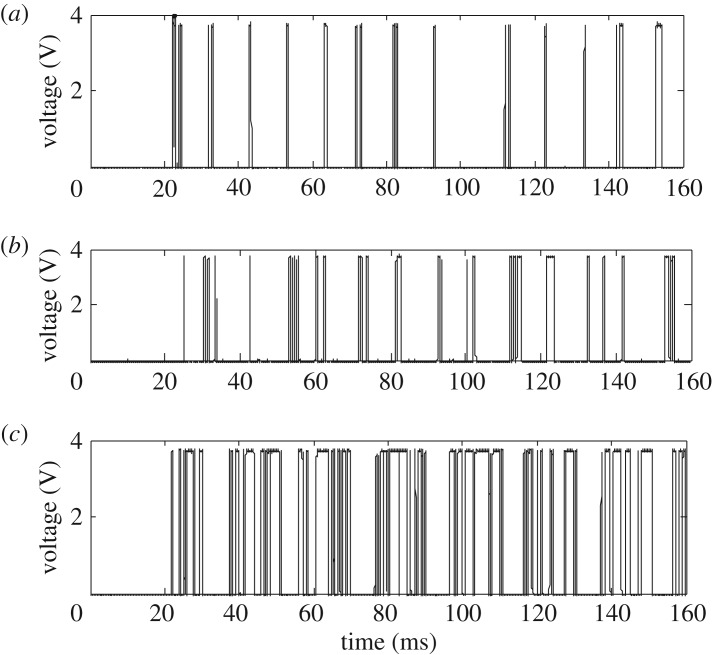
The HFRC extraction results of series arc faults. (*a*) Pulse signal of arc fault under carbonization path suppression, (*b*) pulse signal of arc fault under capacitor filter suppression and (*c*) pulse signal of arc fault under line impedance suppression.

The load used in our experiments was an electric heater. HFRC signals of series arc faults were collected from the carbonization path, capacitance filter suppression and line impedance suppression samples. The extracted pulse signals are shown in figure 5. These data denote that stable pulse characteristics appear for all types of series arc faults. This means that HFRC can be extracted to identify a variety of series arc faults.

**Figure 6.**
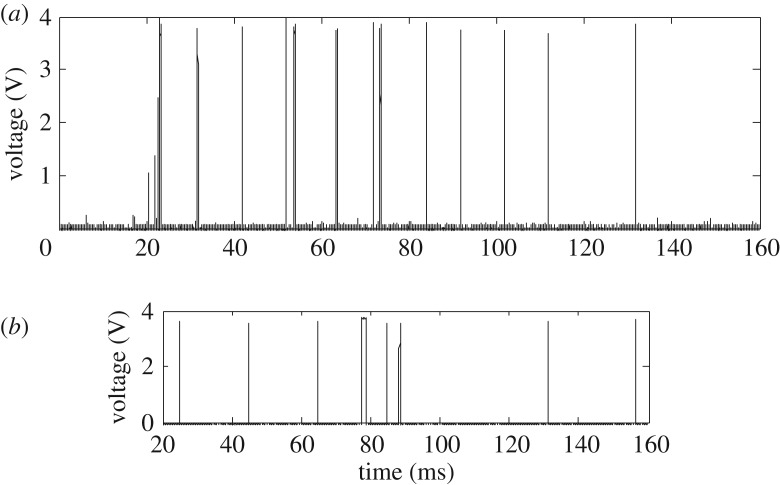
The HFRC extraction results of parallel arc faults. (*a*) The parallel arc faults under carbonization path and (*b*) the parallel arc faults under metal contact.

High-frequency radiation pulse signals of parallel arc faults induced by the carbonization path and the metal contact are shown in figure 6. These data show that the HFRC of the parallel arc faults generated by the carbonization path is clearly more pronounced than that generated by the metal contact. A metal-contact parallel arc fault is produced when a blade cuts two parallel multicore copper wires while they are in use. It is similar to an intermittent short circuit, and the resulting high-frequency pulses are narrow and sparse. Therefore, forming an effective high-frequency pulse in the half arc period of 10 ms is difficult. Using the high-frequency pulse as a criterion therefore invites signal loss and other characteristics are needed for a truly robust arc detection system. For the parallel arc faults caused by the carbonization path and metal contact, the high-frequency characteristics of the arc faults caused by the metal contact are very weak, and it is easy to lose the arc half wave.

### 3.2. Arc fault identification by the CWSC

**Figure 7.**
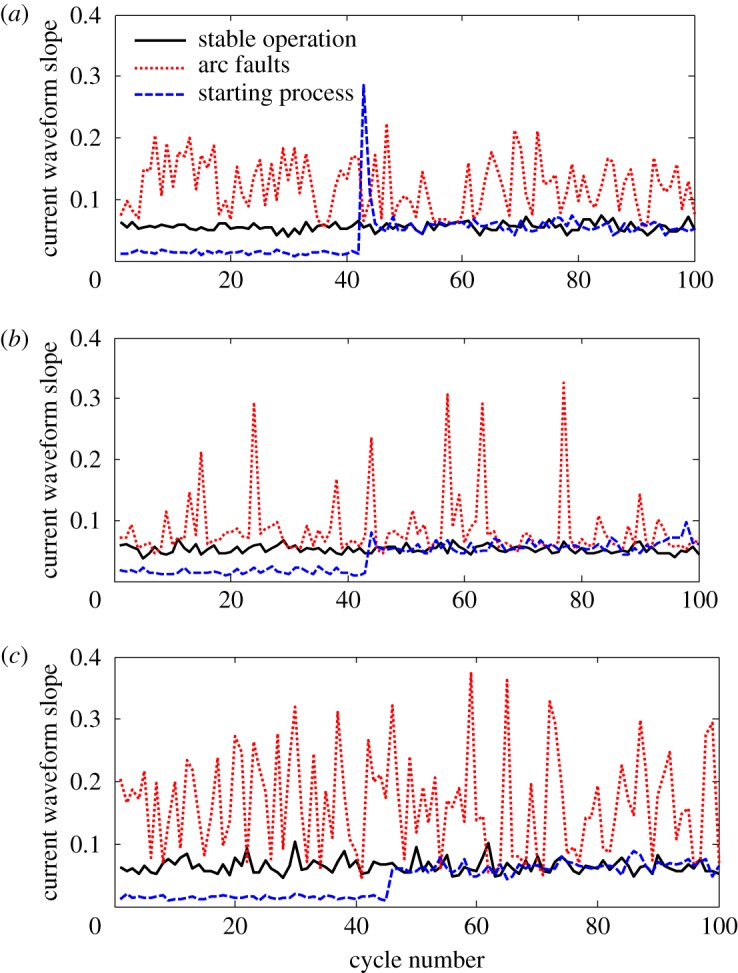
The CWSC of arc faults under single load condition. (*a*) Electric heater load, (*b*) vacuum cleaner load and (*c*) switching power supply.

Figure 7 denotes the CWSC over 1 s measured from circuits with loads of an electric heater, vacuum cleaner and switching power supply during stable operation, start-up and arc faults, as calculated using equation (2.1). When a single load is in stable operation, the CWSC slightly fluctuates. During start-up, the CWSC increases for one or two current half-periods and stabilizes quickly. Therefore, the CWSC detection algorithm will only be slightly affected by the switching on of loads. When arc faults occur in the circuit, the absolute value and rate of change of CWSC therefore increase.

**Figure 8.**
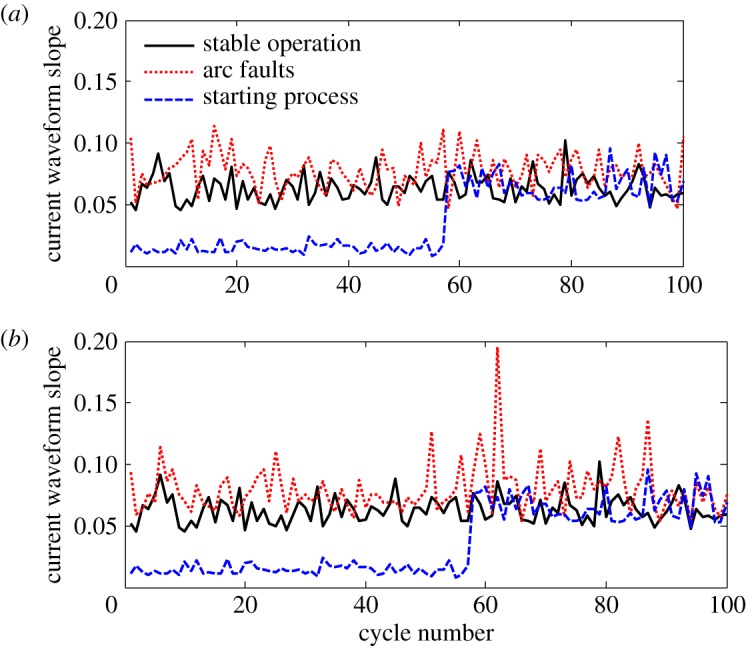
The CWSC of arc faults under combined loads. (*a*) Branch arc fault and (*b*) trunk arc fault.

The CWSC change of the trunk current when the electric heater and switching power supply are connected in parallel was also studied. Figure 8 depicts CWSC changes in the trunk current and the branch current. Figure 8*a* shows CWSC change in the trunk when an arc fault occurs on the branch of the switching power supply. No significant difference between this signal and the normal one was found because of the following reason. When a parallel load on the arc branch is in its normal working state, the calculated current in the denominator of equation (2.1) includes the branch current in this state. When the number of parallel loads increases, the difference between CWSC of the arc fault current waveform and that of the normal waveform decreases. Therefore, detection of CWSC is susceptible to shielded loads. Figure 8*b* shows CWSC changes when an arc fault occurs in the trunk of the circuit. The CWSC is obviously different from the normal one. The main reason is that the statistics are half current waveform of the trunk arc faults. As the CWSC is not increased during each half wave, the difference in slope between each arc current and the normal current is small during each half wave. When the electrical load is simple, the slope of current waveform in series and parallel arc faults is easy to detect. For complex electrical circuits (load includes switching power supply), the branch arc fault recognition algorithm is very susceptible to interference by the slope of current waveform.

### 3.3. Arc fault identification by CWPIC

**Figure 9.**
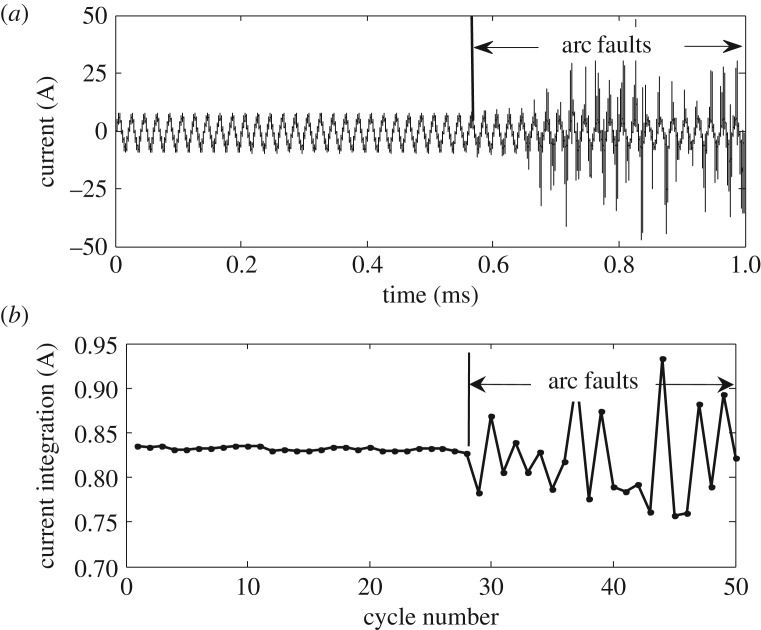
Arc faults of switching power supply branch. (*a*) Time domain waveform of the trunk current and (*b*) periodic integral value change of the trunk current.

When the trunk loads are an electric heater and switching power supply, the arc fault waveforms and CWPIC of the trunk are shown in figure 9. In the stable operation, we see that the trunk current waveforms are stable and the fluctuation of CWPIC is small. When an arc fault occurs on the trunk of the circuit, the amplitude of the CWPIC reduces and random fluctuations appear. The CWPIC variation characteristics are therefore not susceptible to load suppression. However, the change of load power also causes large fluctuations of the CWPIC, so the influence of load-power variation cannot be ruled out only on the basis of the current cycle integral value.

The experimental results can be found from the above. CWSC, CWPIC and HFRC can be used as criteria for identifying arc faults for specific conditions. However, the electrical circuit and working conditions are very complicated. Using one of the criteria to identify arc faults is very likely to lead to misjudgement or leakage. MIF of these three features can improve the accuracy of arc fault recognition algorithm.

### 3.4. Arc fault recognition results

The identification results of arc faults are shown in tables 1 and 2. Outputs close to 1 indicate arc faults and those close to −1 indicate normal operation. The results for a single load in three states are shown in table 1. HFRC, CWSC, CWPIC and the fusion feature of these three signals are used as arc fault criteria. On three operating conditions, the arc fault identification results for each feature are shown in table 1. For a single load loop, the series arc faults can be effectively detected by HFRC. But, some parallel arc faults are prone to leak detection by only HFRC. When the load is induction cooker and switching power supply, stable operation and start-up cannot be separated from arc faults by CWSC. Start-up of load is easily considered as arc faults by CWPIC. Under different working conditions and loads, arc faults can be identified accurately by MIF. These results show that our algorithm can accurately identify the arc faults with a single load. To prove the usefulness of the algorithm, the states of stable operation, start-up, branch arc and trunk arc for different load combinations were also detected. These detection results are shown in table 2. When the load is a combination of electric heater and switching power supply, trunk arc faults cannot be identified by HFRC. The start-up of the load is misjudged to be arc faults by CWSC. When the load is a combination of electric heater and vacuum cleaner, the start-up of the load is misjudged to be arc faults by CWPIC. Arc faults in vacuum cleaner branch and trunk had not been identified by CWPIC.

**Table 1.** Arc fault identification results of single load.

load	state	identification results			
		HFRC	CWSC	CWPIC	MIF
hair drier	stable operation	−1	−1	−1	−1
	start-up	−1	−1	1	−1
	arc faults	1	1	1	1
electric heater	stable operation	−1	−1	−1	−1
	start-up	−1	−1	1	−1
	arc faults	1	1	1	1
vacuum cleaner	stable operation	−1	−1	−1	−1
	start-up	−1	−1	1	−1
	arc faults	1	1	1	1
electric hand drill	stable operation	−1	−1	−1	−1
	start-up	−1	−1	1	−1
	arc faults	1	1	1	1
halogen lamp	stable operation	−1	−1	−1	−1
	start-up	−1	−1	−1	−1
	arc faults	1	1	1	1
induction cooker	stable operation	−1	1	−1	−1
	start-up	−1	−1	1	−1
	arc faults	1	1	1	1
microwave oven	stable operation	−1	−1	−1	−1
	start-up	−1	−1	1	−1
	arc faults	1	1	1	1
switching power supply	stable operation	−1	1	−1	−1
	start-up	−1	−1	−1	−1
	arc faults	1	1	1	1
dimming lamp	stable operation	−1	−1	−1	−1
	start-up	−1	−1	1	−1
	arc faults	1	1	1	1

**Table 2.** Arc fault identification results of combination loads.

load	state	identification results			
		HFRC	CWSC	CWPIC	MIF
electric heater and switching power supply	stable operation	−1	−1	−1	−1
	start-up	−1	1	−1	−1
	arc faults in electric heater branch	1	1	1	1
	arc faults in switching power supply branch	1	1	1	1
	trunk arc faults	−1	1	1	1
electric heater and vacuum cleaner	stable operation	−1	−1	−1	−1
	start-up	−1	−1	1	−1
	arc faults in electric heater branch	1	1	1	1
	arc faults in vacuum cleaner branch	1	1	−1	1
	arc faults in trunk	1	1	−1	1

These results show that the output values of this recognition algorithm are −1 when the circuit is in stable operation or start-up for a single load and a combined load by the MIF of HFRC, CWSC and CWPIC. All arc faults can be accurately identified by MIF. This indicates that the algorithm can detect arc faults through the trunk when each branch load is different.

## 4. Conclusion

If an algorithm relies upon a single signal feature to detect arc faults under different loads, signal loss and cross-talk often occur. We developed a signal-processing method that extracts HFRC, CWSC and CWPIC of arc faults. Then, we designed a recognition algorithm of arc faults with SVM and MIF to remove the interference of different loads in the stable operation and the start-up processes. We found that the algorithm can effectively detect arc faults without signal loss or cross-talk in series circuits with the single and the combined loads, as well as in parallel circuits with a metal contact and a carbonizing path. This recognition algorithm of arc faults can be used to develop arc fault circuit interrupters for circuit branches that can detect arc faults more accurately. The major conclusions are summarized as follows:

(1) Under different load conditions, the HFRC of arc faults changed greatly. The combination of logarithmic detection and linear amplification can be used to stabilize the HFRC extraction. The identification of arc faults by HFRC can easily lead to leakage or misjudgement.
(2) The changes of CWSC are an effective criterion for the arc faults, but this parameter is easily affected by the shielding of the restraining load. It is very difficult to determine the accurate selection of the threshold.
(3) The CWPIC of arc faults can be used as an identification criterion, but this criterion is easily disturbed by the change of load power.
(4) Under complex conditions, the MIF can improve the recognition accuracy of arc faults. The SVM model of arc faults avoids leakage and misjudgement. This recognition algorithm lays the foundation for the development of arc fault circuit interrupters.

## Supplementary Material

Fig.3_data

## Supplementary Material

Fig.4_data

## Supplementary Material

Fig.5_data

## Supplementary Material

Fig.6_data

## Supplementary Material

Fig.7_data

## Supplementary Material

Fig.8_data

## Data accessibility

We uploaded the experimental data as electronic supplementary material.
